# Topical Application of PPADS Inhibits Complement Activation and Choroidal Neovascularization in a Model of Age-Related Macular Degeneration

**DOI:** 10.1371/journal.pone.0076766

**Published:** 2013-10-09

**Authors:** Kerstin Birke, Erion Lipo, Marco T. Birke, Rajendra Kumar-Singh

**Affiliations:** Department of Ophthalmology, Tufts University School of Medicine, Boston, Massachusetts, United States of America; University of Utah (Salt Lake City), United States of America

## Abstract

Age-related macular degeneration (AMD) is the most common cause of blindness among the elderly. AMD patients have elevated levels of membrane attack complex (MAC) in their choroidal blood vessels and retinal pigment epithelium (RPE). MAC forms pores in cell membranes. Low levels of MAC result in an elevation of cytokine release such as vascular endothelial growth factor (VEGF) that promotes the formation of choroidal neovascularization (CNV). High levels of MAC result in cell lysis and RPE degeneration is a hallmark of advanced AMD. The current standard of care for CNV associated with wet AMD is intravitreal injection of anti-VEGF molecules every 4 to 12 weeks. Such injections have significant side effects. Recently, it has been found that membrane pore-forming proteins such as α-haemolysin can mediate their toxic effects through auto- and paracrine signaling and that complement-induced lysis is amplified through ATP release followed by P2X receptor activation. We hypothesized that attenuation of P2X receptor activation may lead to a reduction in MAC deposition and consequent formation of CNV. Hence, in this study we investigated topical application of the purinergic P2X antagonist Pyridoxalphosphate-6-azophenyl-2',4'-disulphonic acid (PPADS) as a potential treatment for AMD. We found that 4.17 µM PPADS inhibited formation of HUVEC master junctions and master segments by 74.7%. In a human complement mediated cell lysis assay, 104 µM PPADS enabled almost complete protection of Hepa1c1c7 cells from 1% normal human serum mediated cell lysis. Daily topical application of 4.17 mM PPADS for 3 days attenuated the progression of laser induced CNV in mice by 41.8% and attenuated the deposition of MAC at the site of the laser injury by 19.7%. Our data have implications for the future treatment of AMD and potentially other ocular disorders involving CNV such as angioid streaks, choroidal rupture and high myopia.

## Introduction

Age-related macular degeneration (AMD) is the most common cause of blindness among the elderly, affecting approximately 1 in 3 people over the age of 65 [1]. AMD begins with the appearance of lipoproteinaceous deposits known as *drusen* between the retinal pigment epithelium (RPE) and Bruch’s membrane [2], [3], [4]. This stage of the disease is generally referred to as ‘dry’ AMD. In approximately 10% of patients, dry AMD progresses to the ‘wet’ form of the disease, involving the growth of new blood vessels from the choroidal vasculature into the subretinal space (choroidal neovascularization, CNV). These immature new blood vessels leak fluid, leading to formation of a macular edema (reviewed in [2]). If left untreated, wet AMD leads to degeneration of retinal tissues and eventually blindness.

Factors implicated in AMD include age, oxidative stress, diet, smoking and activation of complement (reviewed in [1]). Through a yet unclear mechanism, these factors can lead to an elevation of vascular endothelial growth factor (VEGF) in the eyes of AMD patients [5], [6], [7]. Hence, wet AMD patients are currently treated with anti-VEGF antibodies including *ranibizumab* (lucentis), *bevacizumab* (avastin) or anti-VEGF receptor *aflibercept* (Eylea) [8], [9]. However, these therapies are administered by direct (intravitreal) injection into the eye. In addition to being uncomfortable, intravitreal injections are associated with retinal detachment, increased intraocular pressure and endophthalmitis [10], [11]. While these risks are relatively low, they accumulate over the lifetime of the patient. Moreover, these therapies are administered every 4 to 12 weeks. This frequency of administration leads to a significant burden for the elderly, leading to a reduction in personal freedom, a reduction in quality of life and subsequent depression [12], [13]. Cumulatively, these factors result in a reduction in patient compliance [12]. Therefore, there is currently an unmet need for the development of a therapy for AMD that can be applied non-invasively and in a manner convenient to patients, i.e. topically.

Although AMD is a complex disease, approximately 50% of the heritability of AMD can be accounted for by a polymorphism in the complement regulator Factor H [14], [15], [16]. Activation of complement terminates upon the formation of the membrane attack complex (MAC)- a pore formed in the membrane that leads to cell lysis [17]. MAC is elevated in the choroidal blood vessels and RPE of AMD patients [18]. A polymorphism in C9 that prevents the complete formation of MAC protects against the onset of wet AMD [19]. Expression of CD59, the major inhibitor of MAC is reduced in the RPE of advanced AMD patients [20]. We have previously shown that expression of CD59 in the eyes of the most commonly used mouse model of CNV leads to a reduction in MAC and a consequent reduction in CNV [21].

Previously, it has been shown that lysis by membrane pore-forming proteins such as α-haemolysin, leukotoxin and α-toxin can mediate their toxic effects through auto- and paracrine signaling [22], [23], [24]. Subsequently, it has been found that complement induced lysis of human erythrocytes is amplified through ATP release followed by P2X receptor activation [25]. Pyridoxalphosphate-6-azophenyl-2',4'-disulphonic acid (PPADS) is a non-selective purinergic P2X antagonist [26], [27]. We wished to test the hypothesis that PPADS is an effective inhibitor of MAC deposition and CNV in a model of wet AMD. In addition, we wished to test the hypothesis that unlike larger molecules such as antibodies, PPADS may be an effective inhibitor of MAC deposition and CNV by *topical* application. To complement our *in vivo* studies, we also wished to examine the effects of PPADS on tube formation by endothelial cells, complement mediated cell lysis and MAC deposition *in vitro*. If successful, our results will have significant implications for the treatment of AMD and perhaps other diseases involving CNV, such as angioid streaks, choroidal rupture or high myopia [28], [29], [30].

## Results

### PPADS inhibits formation of tubes by HUVECs

**Figure 1:**
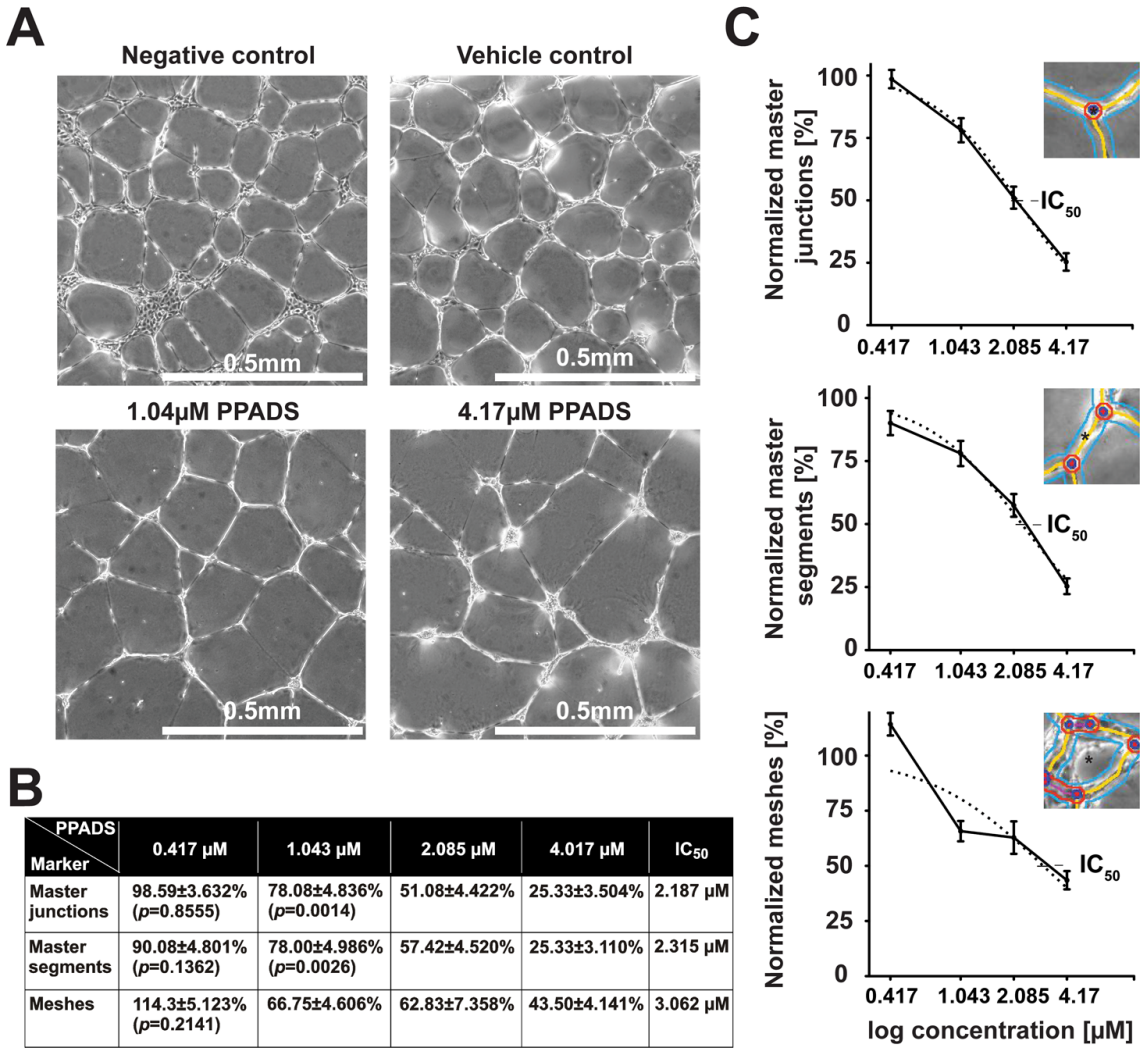
PPADS inhibits formation of tubes by HUVECs. **A**) Representative phase contrast images of tubes formed in the absence of supplements (negative control), in the presence of vehicle (0.9% NaCl) or vehicle plus 1.04 µM PPADS or 4.17 µM PPADS. **B**) Tabular summary of mean values ± SEM of master junctions, master segments and meshes in cells incubated with the indicated concentration of PPADS and calculated IC_50_ for each marker. **C**) Dose dependent PPADS mediated reduction of master junctions, master segments and meshes. The punctuated line indicates the extrapolated inhibition trend. IC_50_ values are indicated as intercept points in the trend line. Asterisks in each inset mark an example of a measured junction, segment or mesh, respectively. Studies were performed a total of 4 times in triplicate.

One commonly accepted model for AMD pathogenesis suggests that prior to the formation of CNV, endothelial cells migrate from the choroid and form tubes (neovascular membranes) in response to elevated cytokines such as VEGF in the intraocular compartment [31]. In order to determine whether PPADS can inhibit endothelial cell tube formation, we performed human umbilical vein endothelial cell (HUVEC) tube formation assays in the presence and absence of PPADS. The ability of HUVECs to form master junctions, master segments or meshes was scored using the ‘HUVEC angiogenesis analyzer’ plugin for Image J Software. We found that the ability of HUVECs to form master junctions, master segments and meshes was significantly reduced with increasing levels of PPADS in a concentration dependent manner (Figure 1A). Specifically, there was a 50% reduction in master junctions, master segments and meshes at 2.187 µM, 2.315 µM, 3.062 µM PPADS respectively (Figure 1B). At higher concentrations of PPADS (4.17 µM) master junctions and master segments were reduced by 74.7% and meshes by 56.5% (Figure 1B, 1C). We conclude that PPADS can inhibit the formation of tubes by HUVECs.

### PPADS Inhibits Complement Mediated Cell Lysis

**Figure 2:**
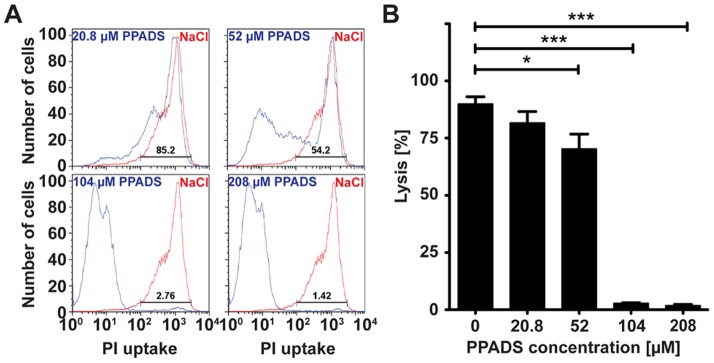
PPADS Inhibits Complement Mediated Cell Lysis. **A**) Representative flow cytometry plots indicating number of Hepa1c1c7 cells taking up propidium iodide (PI) when incubated in 1% normal human serum (NHS) and various concentrations of PPADS (blue lines) or 0.9% NaCl (red lines). **B**) Mean values ± SEM of PPADS mediated reduction of NHS mediated cell lysis from 5 independent studies. Significant differences are shown as capped lines between vehicle control (0.9% NaCl, 0 µM PPADS) and the various concentrations of PPADS (*** p<0.0005, ** p<0.005, * p<0.05).

Activation of complement terminates in the formation of the MAC on the cell surface. Low levels of MAC increase cell mitogenesis and VEGF release [32], [33]. Higher levels of MAC result in cell lysis. Elevated levels of MAC are found on the RPE of AMD patients [18] and RPE loss is a hallmark of advanced AMD known as geographic atrophy. In order to determine whether PPADS can inhibit complement mediated cell lysis, we incubated Hepa1c1c7 cells with 1% normal human serum (NHS) and measured uptake of propidium iodide by FACS. Whereas 1% NHS preconditioned with vehicle lysed 89.98±3.14% of Hepa1c1c7 cells, 1% NHS preconditioned with 104 µM PPADS lysed only 2.88±0.17% (*p*<0.0001) of Hepa1c1c7 cells (Figure 2A). PPADS mediated inhibition of cell lysis was concentration dependent with 20.8 µM and 52 µM PPADS resulting in 81.68±4.97% (*p* = 0.1958) and 70.32±10.57% (*p* = 0.0255) cell lysis respectively (Figure 2B). At 208 µM PPADS, 1.859±0.42% (*p*<0.0001) of Hepa1c1c7 cells were lysed by NHS (Figure 2B). We conclude that PPADS attenuates complement mediated cell lysis.

### PPADS Inhibits Formation of MAC *in vitro*

**Figure 3:**
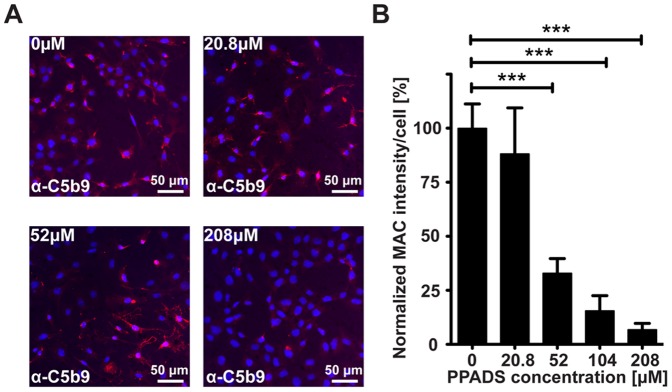
PPADS Inhibits Formation of MAC *in vitro*. **A**) Representative images of C5b9 labeling (red) of cells incubated with increasing concentrations of PPADS. Nuclei are counterstained with DAPI (blue). **B**) Mean values ± SEM of C5b9 staining intensity per cell at various concentrations of PPADS. Values represent cumulative data from 4 independent studies. Significant differences are indicated as capped lines between vehicle control (0.9% NaCl, 0 µM PPADS) and the various concentrations of PPADS (*** p<0.0005, ** p<0.005, * p<0.05).

MAC formation begins with the assembly of the C5b678 complex on the cell surface followed by recruitment of multiple C9 molecules to complete the formation of a pore (C5b9) [32], [34], [35]. In order to determine whether PPADS mediated inhibition of cell lysis correlated with a reduction in the formation of MAC on the cell surface, we incubated Hepa1c1c7 cells in 10% NHS preconditioned with varying concentrations of PPADS and measured levels of C5b9 deposited on the cell surface by immunocytochemistry. We found an inverse correlation between the concentration of PPADS in the media and the amount of C5b9 deposited on the cell surface (Figure 3A). Specifically, when normalized against 0 µM PPADS, 52 µM, 104 µM and 208 µM significantly decreased surface MAC deposition by 67.03±6.81% (*p* = 0.0002), 84.42±16.9% (*p* = 0.0001) and 93.17±11.67% (*p*<0.0001), respectively (Figure 3B). 20.8 µM PPADS did not significantly reduce MAC deposition (*p* = 0.6283, Figure 3B). We conclude that PPADS attenuates formation of MAC *in vitro*.

### PPADS Inhibits Formation of laser induced CNV

**Figure 4:**
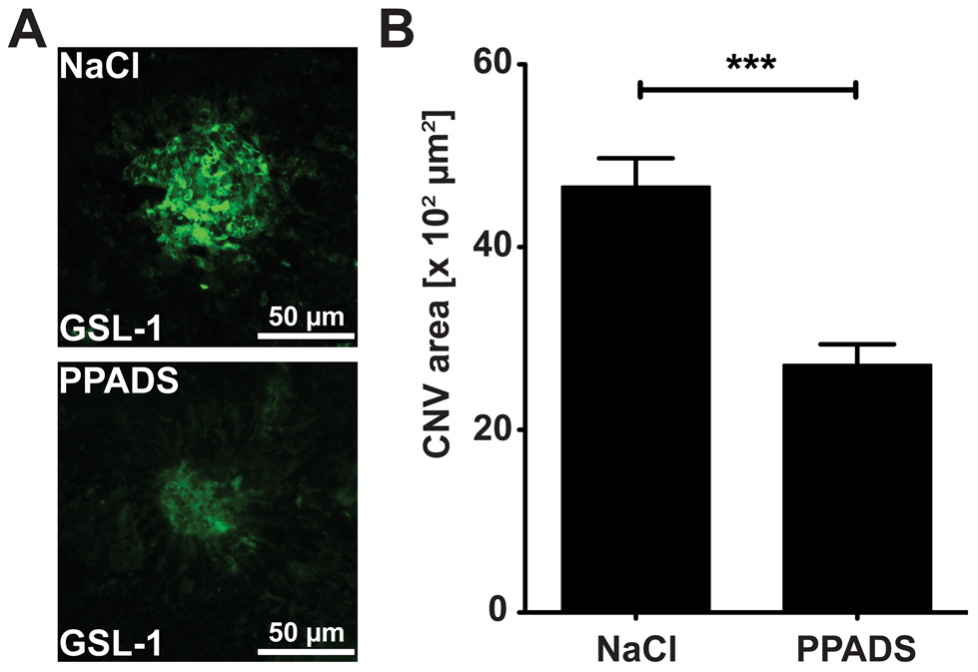
PPADS Inhibits Formation of laser induced CNV. **A**) Representative images of GSL-1 stained CNV at day 7 post laser in a mouse eye treated with 0.9% NaCl or 4.17 mM PPADS topically for 3 days post laser. **B**) Mean values ± SEM of CNV area of 0.9% NaCl treated eyes or 4.17 mM PPADS treated eyes at 7 days post laser. Studies were performed 3 times with 5 mice in each group, n = 15, NaCl = 91 laser spots, PPADS = 71 laser spots (*** p<0.0005).

The most commonly used animal model of wet AMD is laser induced CNV [31]. This model involves a disruption of the Bruch’s membrane, which leads to the rapid generation of CNV [31]. The model responds to a very large variety of inhibitors of angiogenesis and hence while it has limitations, it has become the ‘industry standard’ model for testing the efficacy of molecules intended to attenuate CNV [31]. Most frequently, drugs are injected just prior or immediately after creation of the laser induced rupture of Bruch’s membrane. In our studies, we wished to examine whether topical application of 4.17 mM PPADS immediately after the laser burn followed by daily topical dosing for 3 days would attenuate the progression of CNV. Both eyes of each mouse were treated either with PPADS or buffer NaCl. At the end of the procedure, endothelial cells in the choroidal flat mounts from PPADS or vehicle control mice were stained with GSL-1 lectin (Figure 4A) and quantified using Image J software. We found that PPADS attenuated the area of CNV by 41.8±8.7% (*p<*0.0001, Figure 4B). We conclude that PPADS attenuates endothelial cell migration in laser induced CNV.

### PPADS Inhibits Deposition of MAC at the site of laser induced CNV

**Figure 5:**
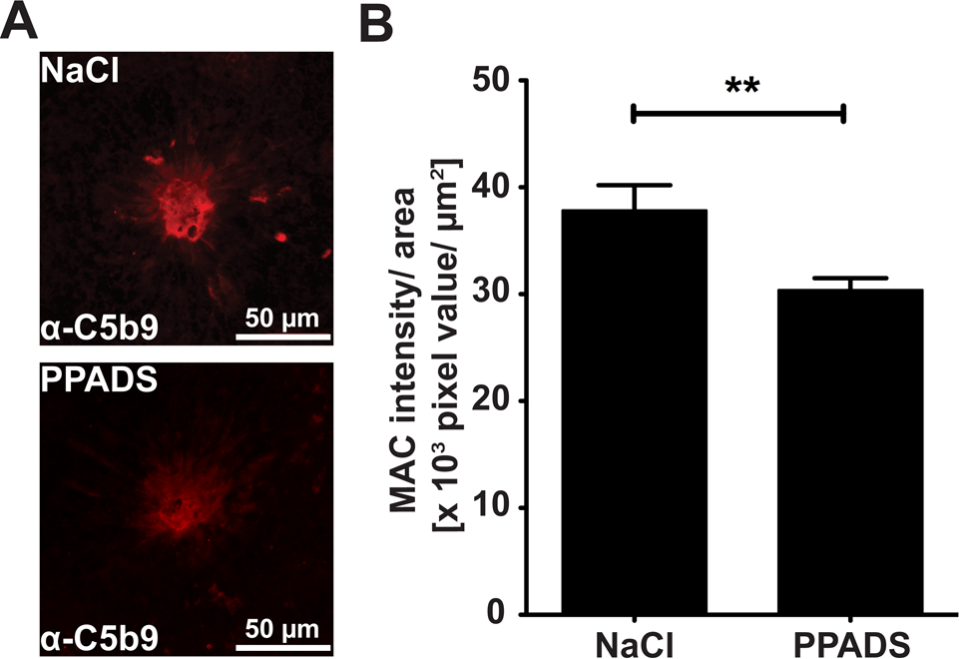
PPADS Inhibits Deposition of MAC at the site of laser induced CNV. **A**) Representative images of C5b9 labeling at 7 days post laser in eyes treated with 0.9% NaCl or 4.17 mM PPADS topically for 3 days. **B**) Mean values ± SEM of C5b9 intensity/area of 0.9% NaCl treated eyes and 4.17 mM PPADS treated eyes 7 days post lasering. Studies were performed 3 times with 5 mice in each group, n = 15, NaCl = 91 laser spots, PPADS = 71 laser spots (** p<0.005).

Recent data indicate that MAC may be a key player in the pathogenesis of AMD [18]. MAC is elevated in the RPE and chroidal blood vessels of AMD patients [18] and inhibitors of MAC are reduced on the surface of cells lost in advanced AMD [20]. An inability to form MAC protects against wet AMD [19] and an inhibition of MAC in animal models of wet AMD attenuates the progression of CNV [21]. Mice were ‘treated’ with PPADS or vehicle control as above and MAC deposition identified using immunohistochemistry against C5b9 (Figure 5A). Area of MAC deposition was quantified using Image J software. We found that PPADS enabled a 19.7±7.5% (*p = *0.0091) reduction in levels of MAC relative to vehicle control (Figure 5B). We conclude that PPADS attenuates MAC deposition in laser induced CNV.

## Discussion

This is the first study examining topical delivery of PPADS as a potential therapy for AMD. We have determined that PPADS can inhibit endothelial tube formation, complement mediated cell lysis and MAC deposition *in vitro* as well as inhibit MAC deposition and laser induced CNV *in vivo*. Our results have potential implications for the future treatment of AMD and possibly other ocular diseases involving CNV, including angioid streaks, choroidal rupture and high myopia.

Our studies were motivated primarily by the current limitations in the treatment procedure utilized for wet AMD patients, which require intraocular injection every 4 to 12 weeks under local anesthesia [8]. Such injections have significant side effects including retinal detachment, an increase in intraocular pressure, endophthalmitis etc. and lead to a large bolus of drug in the intraocular compartment immediately after the injection that dissipates over time [10], [11]. Given that VEGF plays significant roles in maintenance of the retina, these large ‘pulses’ of anti-VEGF molecules are detrimental to tissue homeostasis [36], [37]. Since intravitreal injections are outpatient procedures, the cost to the healthcare system is substantial given the very high prevalence of wet AMD [13]. An alternative mode of drug delivery such as self-administered topical drops is hence potentially very attractive.

Another motivation for our studies was to identify a drug that may inhibit complement activation and specifically reduce MAC deposition. A substantial amount of data is emerging suggesting that an imbalance between complement activation and complement inhibition is responsible for the pathology associated with AMD [14], [16], [38]. A Y402H polymorphism in complement Factor H can explain approximately 43% of AMD cases [16]. AMD patients that are homozygous (HH) have almost 70% higher levels of MAC in their choroid and RPE relative to individuals that are homozygous (YY) for the wild type allele [18]. Inhibitors of complement such as CD46 or more specifically inhibitors of MAC such as CD59 are down regulated in AMD patients [20]. Based on these and other data, at least five inhibitors of complement are currently undergoing clinical trials in the US [39]. Each of these inhibitors is delivered by intraocular or systemic injection. Our studies are the first to demonstrate inhibition of complement activation and specifically a reduction in MAC in any disease model through topical application of a drug. Given the potential role of complement in AMD, it may be envisaged that dry AMD patients could be treated prophylactically with PPADS or an analog of PPADS in order to prevent the progression of dry AMD towards wet AMD.

Several studies have previously examined the potential of topical delivery of drugs for inhibition of CNV [40], [41], [42]. In our studies, we applied PPADS at a concentration of 80 ng once per day for 3 days post laser and found a 40% reduction in CNV at 7 days post laser. Vasostatin, an inhibitor of angiogenesis delivered at 50 ng three times daily for 21 days has been shown to reduce CNV in rats by approximately 50% [40], [43]. Mecamylamine, a nicotinic acetylcholine receptor antagonist has been shown to reduce laser induced CNV by 35% at 100 µg twice a day for seven to 14 days [42]. Aganirsen, an anti-angiogenic aptamer inhibits laser induced CNV almost completely in African green monkeys at 86 µg when applied twice daily for 2 days prior to laser and continued with same regimen for an additional 14 days [41]. In some studies, Pazopanib, a tyrosine kinase inhibitor has been shown to inhibit laser induced CNV in rats but in some other studies no efficacy of Pazopanib has been found [44], [45], [46]. A pazopanib analog, GW771806 is currently undergoing clinical trials [46]. We envisage that PPADS may offer some unique advantages over other molecules, specifically when considering the systems being targeted, i.e. complement activation, MAC deposition as well as angiogenesis.

In this study we provide proof-of-concept for the use of PPADS as an inhibitor of CNV. Future studies will examine the pharmacokinetic, toxicity profile and modes of action of PPADS following topical application to ocular tissues. For now, we hypothesize that in our model of wet AMD, purinergic receptors such as P2X are activated through the release of ATP from cells damaged as a consequence of the laser burn [27]. P2X receptors can also be activated however by oxidative stress, a phenomenon reportedly ongoing in AMD [47], [48]. Activation of purinergic receptors may result in depolarization of cells through Ca^2+^ influx, which may in turn induce expression of additional P2X receptors [27], [49]. Previously, it has been found that intraperitonal injections of suramin or PPADS inhibit oxygen-induced preretinal neovascularization by down-regulating P2X2 receptors in the inner plexiform layer [50]. These collective studies suggest P2X receptor involvement also in retinal disorders. PPADS is classified as a non-hazardous chemical and no detailed toxicity data is currently available. In conclusion, while significant additional studies remain to be performed to identify the mode of action and safety of PPADS as a topical therapy for AMD, here we have provided proof of concept that PPADS or its analogs hold some promise in the treatment of AMD.

## Materials and Methods

Except where indicated, all chemicals and materials were purchased from Fisher scientific company LLC, Suwanee, GA. PPADS was dissolved to a stock concentration of 41.7 mM in 0.9% NaCl.

### Cell culture

Human umbilical vein endothelial cells (HUVEC, Life Technologies Corporation, Carlsbad, CA) were maintained in 200PRF (phenol red free) medium supplemented with 2% Low Serum Growth Supplement (LSGS, Life Technologies Corporation, Carlsbad, CA). Mouse Hepatoma cells (Hepa1c1c7) were purchased from ATCC (Manassas, VA) and maintained in Minimal Essential Medium (MEMα Life Technologies Corporation, Carlsbad, CA) supplemented with 10% fetal bovine serum (FBS). Cells were propagated at 37**°**C in a humidified 5% CO_2_-air incubator.

### Endothelial Tube Formation Assay

Tube formation assays were carried out according to the Endothelial Tube Formation Assay (*In Vitro* Angiogenesis) protocol (Life Technologies Corporation, Carlsbad, CA). Briefly, 9×10^4^ HUVECs were seeded per well on Geltrex™ (Reduced Growth Factor Basement Membrane Matrix, Life Technologies Corporation, Carlsbad, CA) coated wells of a 24-well plate. HUVECs cultured in 200PRF/2% LSGS served as negative controls and vehicle controls contained a total of 0.0045% NaCl. Treatments were performed by adding PPADS to final concentrations of 0.417 µM, 1.043 µM, 2.085 µM and 4.17 µM to 200PRF/2% LSGS medium. After 16 hours the cells were analyzed and images captured with an inverted microscope (IX51; Olympus, Center Valley, PA). Number of master junctions, master segments or meshes in each image were quantified using the “HUVEC angiogenesis analyzer plugin” of ImageJ software.

### Complement Mediated Cell lysis

FACS lysis assays were performed as previously described [21]. Briefly, Hepa1c1c7 cells were grown on T75 cell culture flasks in MEMα containing 2% FBS to 70% confluency. Cells were trypsinized, counted and aliquots of 5×10^5^ cells per assay were incubated in gelatin veronal buffer (GVB) containing PPADS at concentrations of 20.8 µM, 52 µM, 104 µM, 208 µM. Lysis was induced with 1% normal human serum (NHS) for 1 hour at 37°C with continuous gentle rotatory motion. GVB containing a total of 0.225% NaCl, which equals the NaCl content in PPADS containing samples, served as control. Cell lysis was detected as uptake of propidium iodide in 2.5×10^4^ cells by flow cytometry (FACSCalibur, Becton Dickinson). Analysis was done with the FlowJo software (Tree Star Inc., Ashland, OR).

### Membrane Attack Complex (MAC) Deposition Assay

MAC deposition assays were carried out as previously described [21]. In brief, 3×10^4^ Hepa1c1c7 cells were seeded per 8 well chamber slides and grown in MEMα containing 2% FBS for 2 days. Medium was exchanged to GVB/10% NHS containing PPADS at concentrations of 20.8 µM, 52 µM, 104 µM, 208 µM. After incubation for 5 minutes at 37°C, the reaction was terminated with ice-cold PBS and cells fixed in 4% formaldehyde. For MAC detection, cells were blocked in 6% normal goat serum, 0.5% Triton X-100, 1x PBS (blocking solution). MAC was labeled with a mouse anti-C5b9 antibody (ab66768, Abcam, Cambridge, MA diluted 1∶100 in blocking solution) and detected with a Cy3-conjugated goat anti-mouse antibody (Jackson ImmunoResearch Laboratories, West Grove, PA diluted 1∶200 in blocking solution). Nuclei were counterstained with DAPI. Images were captured using an inverted microscope (IX51; Olympus, Center Valley, PA) attached to a fluorescence unit. Integrated Cy3 densities and DAPI signals were quantified using ImageJ software. The amount of MAC deposition was defined as integrated density of C5b9 labeling (Cy3) per cell (DAPI).

### Animals

This study was carried out in strict accordance with the Statement for the Use of Animals in Ophthalmic and Vision Research, set out by the Association of Research in Vision and Ophthalmology (ARVO) and was approved by Tufts University Institutional Animal Care and Use Committee (IACUC) protocol B2011-150 and Tufts University Institutional Biosafety Committee registration 2011-BRIA68. For experiments 6 weeks old C57Bl/6J mice (Jackson laboratory, Bar Harbor, ME) were utilized. Animals were anesthetized by intraperitoneal injections of a mixture containing 0.1 mg/g body weight Ketamine (Phoenix™,St Joseph, MO) and of 0.01 mg/g body weight Xylazine (Lloyed, Shenandoah, Iowa). Mice were kept constantly warm during anesthesia. At indicated time points mice were sacrificed by CO_2_ inhalation, followed by cervical dislocation.

### Laser Induced Choroidal Neovascularization (CNV)

Laser induced CNV was performed as previously described [21]. Briefly, pupils of anesthetized mice were dilated with one drop of 2.5% phenylephrine HCl (Bausch & Lomb Incorporate, Tampa, FL) and one drop of 1% Tropicamide (Bausch & Lomb Incorporate, Tampa, FL). To prevent injuries to the cornea one drop of 2.5% Hypromellolose (Goniovisc, Wellhead, UK) was applied and a cover slip attached. In each eye four laser spots were generated using an argon laser (532 nm, IRIS Medical Light solutions, IRIDEM, IRIDEX, Mountain View, CA) with 100 ms pulse duration and 150 mW power per spot, and a spot size of 75 µm in diameter.

### Topical application of PPADS

A total of 20 µl of a 4.17 mM PPADS solution was topically applied to each eye of anaesthetized mice. Control mice received 20 µl of 0.9% NaCl (vehicle control). Treatment was performed after laser burn for 3 consecutive days in intervals of 24 hours. Mice were sacrificed 7 days after laser, eyes enucleated and passed to histological processing.

### Histological Processing and Immunolabeling

Cornea, iris, lens and the neuronal retina were removed from enucleated eyes. Posterior eye cups were fixed by immersion in 4% PFA and cut four times from the edge to the center to obtain flat mounts. Specimens were blocked in PBS/2.5% BSA and stained with FITC-conjugated Griffonia Simplicifolia Lectin I (GSL-I, Isolectin B4, Vector laboratories, Burlingame, CA) 10 mg/ml in PBS. Flat mounts were washed with PBS, re-blocked with blocking solution (Jackson ImmunoResearch Laboratories, West Grove, PA), incubated with a rabbit anti-human C5b9 antibody (Complement Technology, Inc., Tyler, TX, diluted 1∶200 in blocking solution) and detected with a Cy3-conjugated goat anti-rabbit antibody (Jackson ImmunoResearch Laboratories, West Grove, PA diluted 1∶400 in PBS). Flat mounts were imaged using an inverted microscope (IX51; Olympus, Center Valley, PA) attached to a fluorescence unit and images were captured using a Qimaging RETIGA200R camera (Qimaging, Surrey, BC Canada). Areas of GSL-I labeling (CNV areas) and signal intensities of C5b9 labeled areas (MAC areas) were measured using ImageJ software. Data points for MAC deposition are depicted as C5b9 intensities per C5b9 area ratios. C5b9 intensities were corrected for background intensities before calculations.

### Statistical Analysis

Statistical analysis was performed with the GraphPad5 software (La Jolla, CA). Statistical significance between PPADS treatment groups and control groups were determined by the unpaired student’s t-test. For each *in vivo* experiment, 5 mice per group were utilized. *In vitro* experiments were performed in duplicate. All experiments were repeated at least three times. All data are presented as mean ± SEM.
